# Comparison of the Simplification of the Pressure Profiles Solving the Binary Friction Model for Asymmetric Membranes

**DOI:** 10.3390/membranes7040058

**Published:** 2017-10-03

**Authors:** Unoaku Victoria Unije, Robert Mücke, Stefan Baumann, Olivier Guillon

**Affiliations:** 1 Forschungszentrum Jülich GmbH, Institute of Energy and Climate Research, IEK-1: Materials Synthesis and Processing, D-52428 Jülich, Germany; r.muecke@fz-juelich.de (R.M.); s.baumann@fz-juelich.de (S.B.); o.guillon@fz-juelich.de (O.G.); 2 JARA-Energy, Jülich Aachen Research Alliance

**Keywords:** binary friction model, oxygen transport membrane, porous structure, MIEC, micro porous media, CFD

## Abstract

The gas flow through porous media including that of multiple species is frequently described by the binary friction model (BFM) considering the binary diffusion, Knudsen diffusion, and viscous flow. Therefore, a numerical simulation was performed on a microporous support of an asymmetric oxygen transport membrane. As its exact numerical solution is complicated and not always possible, the results of two different levels of simplification of the pressure profiles within the porous support are compared to the exact numerical solution. The simplification using a constant pressure equal to the gas pressure outside the support leads to a deviation by up to 0.45 mL·min^−1^·cm^−2^ from the exact solution under certain operating condition. A different simplification using a constant pressure averaged between the outside of the support and the support/membrane interface reduces this deviation to zero. Therefore, this is a useful measure to reduce computational efforts when implementing the Binary Friction Model in computational fluid dynamics simulations.

## 1. Introduction

Porous materials are encountered in a wide variety of technologies, where they have been employed for filtration, mixing or reacting transported species, and separation purposes. The description of the transport process of multicomponent or single component fluid through porous media (from nano porous to micro porous media) is of great interest for the modelling of a wide range of processes. Numerous studies have been carried out in the past using Dusty gas model (DGM) [1,2] for the description of the transport through the porous media, but Kerkhoff [3] proved two decades ago that the dusty gas model is invalid because of the errors found in the derivation, where the viscous flux was considered twice. He derived the binary friction model (BFM) starting from the Light foot equation [4], and it combines bulk diffusion, Knudsen diffusion, and viscous flow.

The analytical description of the transport through the porous support using the binary friction model (Equation (1)) according to Kerkhoff [3] has been observed to be sufficient [5,6,7,8,9].

(1) $$\frac{1}{P_{t}}\vec{\nabla }P_{i}=RT\sum_{j=1}^{n}\frac{\left(x_{i}{\vec{N}}_{j}-x_{j}{\vec{N}}_{i}\right)}{P_{t}D_{ij}}-r_{im}{\vec{N}}_{i}$$

where R is the molar gas constant, *T* the temperature, *P_t_* and *P_i_* the total and partial pressure of the diffusing species, respectively, *r_im_* the friction term, and *D_ij_* the effective binary diffusion coefficient. *N*, *x*, and *i* denote the flux, mole fraction, and transported species, respectively.

Mixed ionic and electronic conducting (MIEC) ceramic gas separation membrane [10,11,12] is an example of an applied case for the separation process. Research studies over the years have found that the thinner the dense MIEC ceramic membrane, the higher the observed flux, but the lower the mechanical stability. This motivated the state of the art processing of an asymmetric membrane whereby the thin dense membrane is supported by a porous structure. Asymmetric membranes provide a low ionic resistance of the functional separation layer together with a high mechanical stability. The Wagner equation describes the solid state transport through the dense membrane. A modification of this equation as the thickness of the dense membrane is reduced to increase flux considers the surface exchange effect by the introduction of the characteristic thickness, *L_c_* [13].

(2) $$N_{O_{2}}=-\frac{RT}{16F^{2}}\cdot \frac{1}{L+2L_{c}}\cdot \sigma_{amb}\cdot \ln\frac{{p^{\prime }}_{O_{2}}}{{p^{\prime \prime }}_{O_{2}}}$$

where *N*_O_2__, R, *F*, *T*, *σ_amb_*, and *P*_O_2__, are the molecular flux, molar gas constant, Faraday’s constant, temperature, ambipolar conductivity, and oxygen partial pressures at both sides of the membrane, respectively. *L* is the membrane thickness and *L_c_* represents the thickness at which the oxygen transport changes from bulk diffusion controlled to surface exchange kinetics controlled. The influence of the porous support on flux [5,6,7] through an asymmetric membrane cannot be over emphasized. As a result, there is a need to optimize the porous structure to provide the necessary mechanical stability with minimal or no limitation on the observed flux.

The relationship between the experimental and simulated flux through an asymmetric Ba_0.5_Sr_0.5_Co_0.8_Fe_0.2_O_3–δ_ membrane using a 1D BFM for the transport through the porous support has been presented in previous work [14]. Li et al. [6] simulated the effect of the porous support of an asymmetric membrane on flux by coupling a 2D fluent to the BFM and the Wagner equation to describe the transport through a supported membrane. The Wagner equation (Equation (2)) and the BFM were used for the description of the transport through the dense membrane and the porous support, respectively. They observed that the H_2_ and CO_2_ fluxes through the membrane declined by 10.3–18.8% and 0.6–1.5%, respectively, because of the porous support.

Computational fluid dynamics (CFD) is an important tool that has contributed greatly to the development of membranes [15,16,17,18], and is suitable for simulating numerically the solution of the BFM in 3D. However, the exact solution of the BFM for the 3-dimensional numerical description of the transport through the porous support requires high computational efforts. The 1D analytical simulation of the transport through the porous support using the exact solution of the BFM in Mathematica takes approximately 10s on the current work station. This will increase drastically if the BFM is solved locally for a realistic 3D microstructure. As this requires solving for the millions of cells inside the support. Therefore, evaluation of different simplification approaches of the pressure profiles inside the porous body motivated this work.

In the present study the BFM and the Wagner equation [3,13] were used to describe in 1D the transport through the porous support and the dense membrane, respectively. The description of the transport through the porous support by the BFM was considered using the exact solution and two simplifications with respect to the pressure profile inside the porous support, which is non-linear. The simplifications considered are (i) surface constant pressure and (ii) an averaged constant pressure. The BFM has been reported in literature assuming an averaged pressure simplification [6], but in this work, an in-depth study of the comparison between the deviation of the constant pressure simplifications and the exact solution were studied, and the implication/effect of each simplification on the observed flux, are presented. The outcome of the comparison will make the implementation of the BFM for 3-dimensional CFD simulation less time consuming and computationally less expensive.

## 2. Procedure

### 2.1. Transport Modes

**Figure 1:**
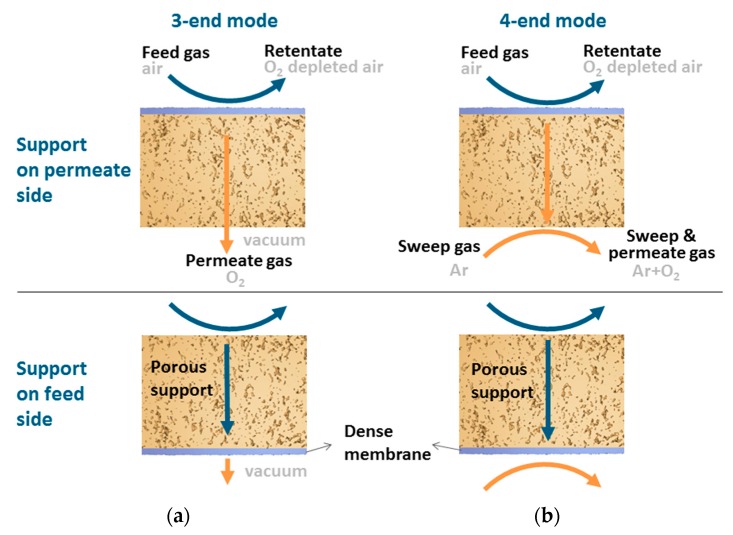
The (**a**) 3-end mode and (**b**) 4-end mode transport processes, with the support at the feed or permeate side, respectively.

The modes of transport employed in the present work are the 3-end mode (Figure 1a) and 4-end mode (Figure 1b) of transport depending on how the permeated gas is removed or collected. For the 4-end mode transport process, a sweep gas is used to remove the permeated gas, whereas, for the 3-end mode, a vacuum is used for the removal of the permeated gas. The same oxygen partial pressures for the feed and permeate side were employed for the different operating conditions. The abbreviation SF and SP will be used in the rest of the paper to show when the support is at the feed and permeate side, respectively.

### 2.2. Fundamental Equation

Air was used as the feed gas, as such *n* = 2, *i =* 1 ≡ O_2_, and *i =* 2 ≡ Ar/Nitrogen, with the flux N_2_ = 0.

Using the ideal gas equation, the mole fraction of the species in the BFM (Equation (1)) was substituted using Equation (3)

(3) $$x_{i}=\frac{P_{i}}{P_{t}}$$

Also, the friction term [3,14] for the single gas Equation (4)

(4) $$\frac{1}{r_{gm}}=\frac{P_{g}}{RT}\left(D_{g}^{K}+\frac{B_{o}P_{g}}{\eta_{g}}\right)$$

and for the mixed gas permeation Equation (5) were substituted.

(5) $$r_{im}=\frac{RT}{P_{t}}{\left(D_{i}^{K}+\frac{B_{o}P_{t}}{\eta_{i}}\right)}^{-1}$$

The flux of oxygen through the support can be obtained from the BFM for single gas and mixed gas permeation using:

(6) $$N_{g}=-\frac{\nabla P_{g}}{RT}\left(\frac{\varepsilon }{\kappa }D_{g}^{K}+\frac{B_{o}P_{g}}{\eta_{g}}\right)$$

and,

(7) $$N_{1}=-\frac{\nabla P_{1}}{RT}\frac{1}{\underset{\begin{array}{l} \mathrm{Binary}\\ \mathrm{diffusion} \end{array}}{\underset{︸}{\frac{P_{t}-P_{1}}{\frac{\varepsilon }{\kappa }D_{12}P_{t}}}}+\frac{1}{\underset{\begin{array}{l} \mathrm{Knudsen}\\ \mathrm{diffusion} \end{array}}{\underset{︸}{\frac{\varepsilon }{\kappa }D_{1}^{K}}}+\underset{\begin{array}{l} \mathrm{Viscous}\\ \mathrm{flow}\; \end{array}}{\underset{︸}{\frac{B_{o}P_{t}}{\eta_{1}}}}}}$$

respectively. Where R is the molar gas constant, *T* the temperature, *P_t_* and *P_i_* the total and partial pressure of the diffusing species, respectively, *N* the flux, and *D_ij_* the effective binary diffusion coefficient. *B*_o_, η, ε, and κ, are the permeability, fluid viscosity, porosity, and tortuosity factor, respectively.

### 2.3. The Different Pressure Configurations Considered

#### 2.3.1. One Dimensional Exact Solution of the BFM

The total and the partial pressures are considered to vary through the position *X* along the support thickness. The exact solution gives a more realistic description of the transport through the porous support. The single and mixed gas equations for the exact solution of the BFM (Equations (6) and (7)) are: 

For single gas permeation

(8a) $$N_{g}=-\frac{\nabla P_{g}(X)}{RT}\;\underset{\begin{array}{l} \mathrm{Knudsen}\\ \mathrm{diffusion} \end{array}}{\underset{︸}{\frac{\varepsilon }{\kappa }D_{g}^{K}}}+\underset{\begin{array}{l} \mathrm{Viscous}\\ \mathrm{flow} \end{array}}{\underset{︸}{\frac{B_{o}P_{g}(X)}{\eta_{g}}}}$$

For mixed gas permeation

(8b) $$N_{1}=-\frac{\nabla P_{1}(X)}{RT}.\frac{1}{\underset{\begin{array}{l} \mathrm{Binary}\\ \mathrm{diffusion} \end{array}}{\underset{︸}{\frac{P_{t}(X)-P_{1}(X)}{\frac{\varepsilon }{\kappa }D_{12}P_{t}(X)}}}+\frac{1}{\underset{\begin{array}{l} \mathrm{Knudsen}\\ \mathrm{diffusion} \end{array}}{\underset{︸}{\frac{\varepsilon }{\kappa }D_{1}^{K}}}+\underset{\begin{array}{l} \mathrm{Viscous}\\ \mathrm{flow} \end{array}}{\underset{︸}{\frac{B_{o}P_{t}(X)}{\eta_{1}}}}}}$$

The implementation of this model for 3-dimensional CFD simulation is complicated, because the exact solution of the BFM in 3D is a complex numerical solution and requires high computational effort. Therefore, a simplified modification of the BFM with negligible deviation from the outcome of the exact solution is desirable.

#### 2.3.2. BFM–Constant Pressure Configuration

In this pressure simplification, the pressure through the porous support is assumed to be constant. A constant gradient will yield a zero flux; as a result, the pressure gradient (∇*P_i_*) of the diffusing species in the BFM which is the driving force for the transport is assumed to have a linear behavior. This constant pressure simplification is subdivided into surface constant pressure and averaged constant pressure.

##### Surface Constant Pressure

**Figure 2:**
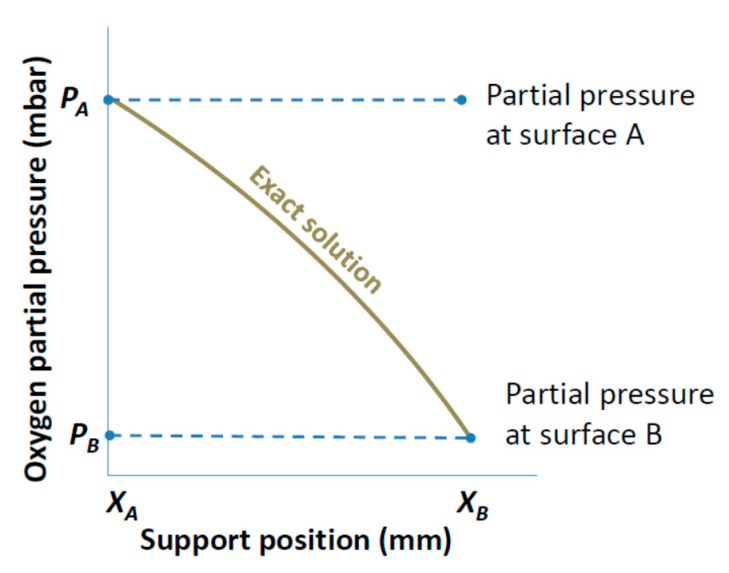
The plot of the exact solution and the surface constant pressure simplification. For the exact solution, *P_A_* and *P_B_* denote the feed and interface or interface and permeate pressure for when the support is at the feed or permeate side, respectively. Whereas for the surface constant pressure simplification, *P_A_* or *P_B_* denote the surface pressure of the diffusing species when the support is at the feed or permeate side, respectively, and this is assumed constant through the support.

In this simplification, the pressure of the free surface of the porous support is assumed to be constant through the porous support. The assumed pressure is dependent on the position of the porous support. If the support is at the feed side, the feed pressure is assumed to be constant through the porous support. Similarly, if the support is at the permeate side, the permeate pressure is assumed to be constant through the porous support. This simplification is graphically shown in Figure 2.

Figure 2 shows the pressure profile through the support for the surface constant pressure simplification. For the evaluation of flux through the asymmetric membrane for this simplification, the single gas and mixed gas permeation equations are same with Equations (6) and (7), respectively.

##### Averaged Constant Pressure

In this pressure simplification, the pressure on both sides of the porous support were averaged and assumed to be constant through the porous support. The feed and interface pressure or the interface and permeate pressure were averaged for the support at the feed side or permeate side, respectively.

**Figure 3:**
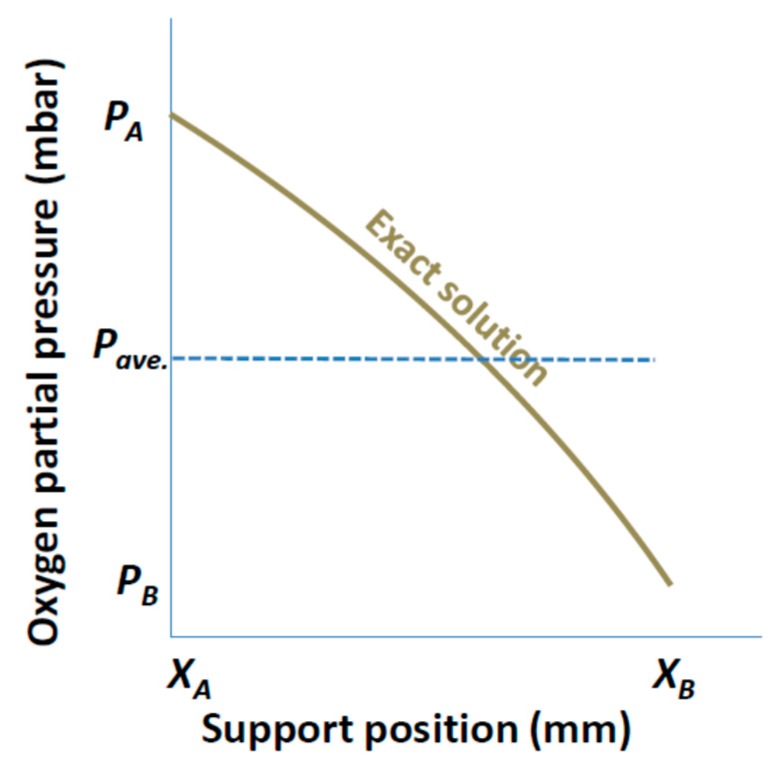
The plot of the exact solution and the averaged constant pressure simplification. For the exact solution, *P_A_* and *P_B_* denote the feed and interface or interface and permeate pressure of the diffusing species when the support is at the feed or permeate side, respectively. Whereas for the averaged constant pressure simplification, the pressure through the support thickness was assumed to be the average of the pressures on both surfaces of the porous support. *P_A_*/*P_B_* is the feed/interface pressure of the diffusing species when the support is at the feed side or interface/permeate pressure when the support is the permeate side.

Depending on the position of the porous support, *P_A_* and *P_B_* can either be feed and interface pressures or interface and permeate pressures, respectively, as can be seen in Figure 3. These pressures (*P_A_* and *P_B_*) are to be averaged and assumed constant through the support. The single and mixed gas permeation equations for averaged constant pressure simplification are:

For single gas permeation

(9a) $$N_{g}=-\frac{\nabla P_{g}}{RT}\underset{\begin{array}{l} \mathrm{Binary}\\ \mathrm{diffusion} \end{array}}{\underset{︸}{\frac{\varepsilon }{\kappa }D_{g}^{K}}}+\;\underset{\begin{array}{l} \mathrm{Viscous}\\ \mathrm{flow} \end{array}}{\underset{︸}{\frac{B_{o}\frac{P_{A}+P_{B}}{2}}{\eta_{g}}}}\;$$

For mixed gas permeation

(9b) $$N_{1}=-\frac{\nabla P_{1}}{RT}\frac{1}{\underset{\begin{array}{l} \mathrm{Binary}\\ \mathrm{diffusion} \end{array}}{\underset{︸}{\frac{P_{t}-\left(\frac{P_{1A}-P_{1B}}{2}\right)}{\frac{\varepsilon }{\kappa }D_{12}P_{t}}}}+\frac{1}{\underset{\begin{array}{l} \mathrm{Knudsen}\\ \mathrm{diffusion} \end{array}}{\underset{︸}{\frac{\varepsilon }{\kappa }D_{1}^{K}}}+\underset{\begin{array}{l} \mathrm{Viscous}\\ \mathrm{flow} \end{array}}{\underset{︸}{\frac{B_{o}P_{t}}{\eta_{1}}}}}}$$

From the BFM equation for single gas permeation, the *P_A_* and *P_B_* are as explained for Figure 3. For the mixed gas permeation equation where there exists more than one gas, *P*_1_*_A_* and *P*_1_*_B_* mean the same as *P_A_* and *P_B_* for the diffusing species of the mixture.

### 2.4. Simulation Procedure

The BFM was used for the description of the transport through the porous support and the Wagner equation for the solid state transport through the dense membrane. Continuity relation was assumed (i.e., the flux through the dense membrane equals that through the porous support) for the evaluation of the flux through the asymmetric membrane. The transport governing terms in the binary friction model are shown in Table 1.

In addition to the transport governing terms in Table 1, the input data in Table 2 being the experimental data reported in references [5] and [13] were employed for the present evaluation.

## 3. Results and Discussion

**Figure 4:**
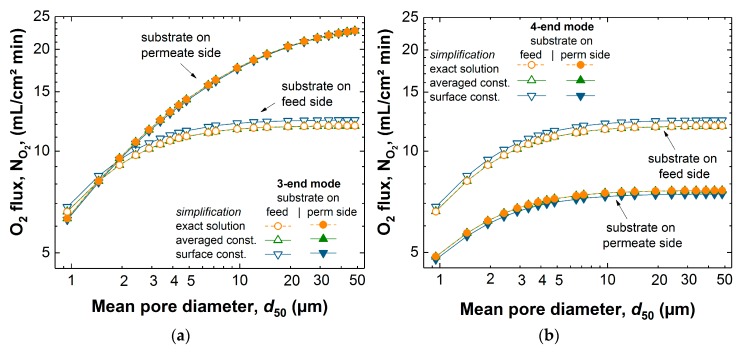
The dependence of flux on the pore diameter of the porous support for (**a**) 3-end and (**b**) 4-end mode of transport, using air as feed gas for the exact solution and the different pressure simplifications (averaged constant and surface constant pressure) of the BFM.

In the earlier work [14], the exact solution of the BFM was used in one dimension to reproduce experimental behavior. In the present study, a slight variation between the different simplifications and the exact solution exists. These variations in flux at varying pore diameter of the porous support from 1 to 50 µm are evident from Figure 4. For all simplifications with the support at the feed side, the 3-end mode and 4-end mode transport through the porous support was described similarly, because the mixed gas permeation equation of the BFM with the support on the feed side was employed for both cases. For 3-end mode transport process with the support at the permeate side, it is a single gas permeation through the porous support. The 1D analytical simulation of the surface constant and averaged constant pressure simplifications for BFM took 0.20 and 0.27 s, respectively. This is more than one order of magnitude faster than that of the exact solution. The flux observed for the exact solution and the simplifications for the transport modes were similar. For mixed gas permeation through the porous support, there was a deviation between the exact solution and simplified solutions.

The maximum deviations for the range of pore diameters considered between the surface constant simplification and the exact solution for the 3-end/4-end mode with the support at the feed side, the 3-end mode with the support at the permeate side, and 4-end mode with the support at the permeate side, are 3.9%, 1%, and 2.5%, respectively. While for the averaged constant pressure simplification, the maximum deviation for all transport modes and configurations in comparison to the exact solution was not more than 0.1%. For the case of the 3-end mode with the support on the permeate side, there was zero deviation. The surface constant pressure simplification led to a larger deviation in comparison to the exact solution than the averaged constant pressure simplification. The surface constant pressure simplification is always off even at small pore diameters (a bit less than at higher pore diameters). Averaged constant pressure simplification is always very close to the exact solution.

Mixed gas permeation has several practical applications (e.g., membrane reactor); therefore, it is important to ascertain clearly how each of the simplifications can be compared to each other and to the exact solution. Therefore, while varying the transport terms (the viscous flow, the binary and Knudsen diffusion) of the BFM, maps of the relative and the absolute difference between the exact and simplified solutions were made.

**Figure 5:**
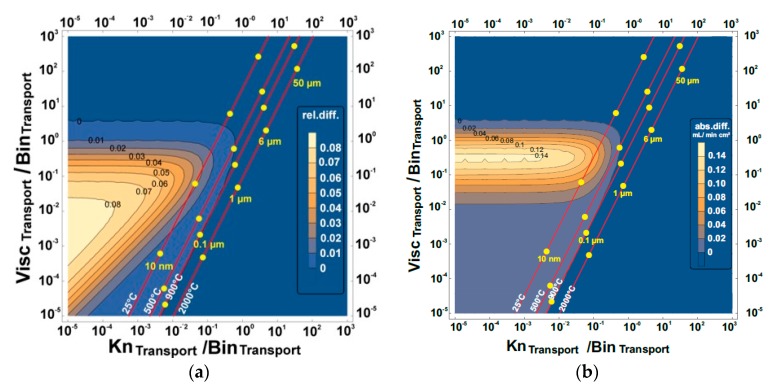
The (**a**) relative and (**b**) absolute difference between averaged constant pressure simplification and the exact solution for the 3-end mode and 4-end mode transport processes with the support at the feed side using air as the feed gas. Visc_Transport_, Bin_Transport_, and Kn_Transport_ represent viscous flow, binary diffusion, and Knudsen diffusion transport, respectively. The red lines are the temperature increase from left to right, while the pore diameter increases upwards.

**Figure 6:**
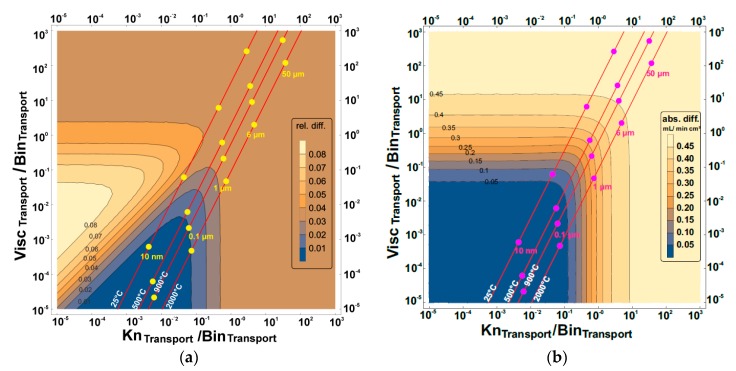
The (**a**) relative and (**b**) absolute difference between the surface constant pressure simplification and the exact solution for the 3-end mode and 4-end mode transport processes with the support at the feed side using air as the feed gas. Visc_Transport_, Bin_Transport_, and Kn_Transport_ represent viscous flow, binary diffusion, and Knudsen diffusion transport, respectively. The red lines are the temperature increase from left to right, while the pore diameter increases upwards.

**Figure 7:**
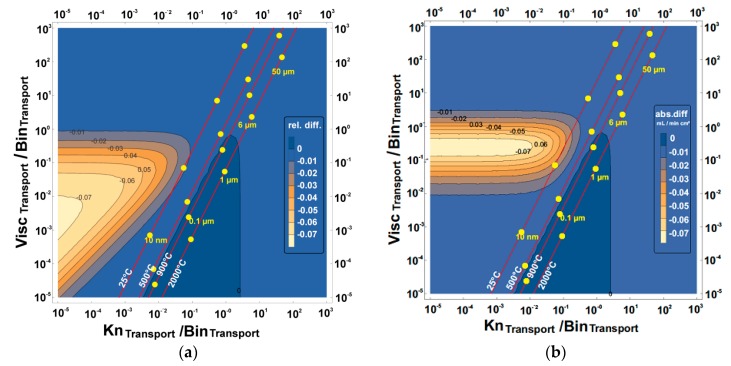
The (**a**) relative and (**b**) absolute difference between averaged constant pressure simplification and the exact solution for the 4-end mode transport process with the support at the permeate side using air as the feed gas. Visc_Transport_, Bin_Transport_, and Kn_Transport_ represent viscous flow, binary diffusion, and Knudsen diffusion transport, respectively. The red lines are the temperature increase from left to right, while the pore diameter increases upwards.

**Figure 8:**
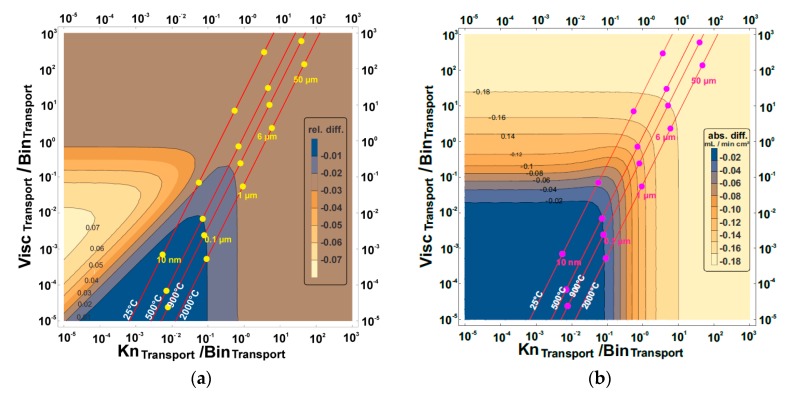
The (**a**) relative and (**b**) absolute difference between surface constant pressure simplification and the exact solution for the 4-end mode transport process with the support at the permeate side using air as the feed gas. Visc_Transport_, Bin_Transport_, and Kn_Transport_ represent viscous flow, binary diffusion, and Knudsen diffusion transport, respectively. The red lines are the temperature increase from left to right, while the pore diameter increases upwards.

These maps are presented in Figure 5, Figure 6, Figure 7 and Figure 8 and show the effect of increasing or reducing each of the transport governing terms of the BFM for the different pressure simplifications in comparison to that of the exact solution. The map was studied to know which of the terms and at what configuration is more rate-limiting. These effects were studied by varying temperature, pore diameter, and tortuosity factor. Except for the Knudsen diffusion, the transport equations (the viscous flow and the binary diffusion) of the BFM were modified for the different pressure simplifications. The Knudsen diffusion has a linear dependence on the mean pore diameter of the porous support, while the permeability is linearly dependent on the square of the mean pore diameter ($B_{o}\propto d_{\mathrm{pore}}^{2}$) of the porous support. The binary diffusion shows no dependence on pore diameter. Air was the only feed gas used for the present evaluation. As such, only the mixed gas permeation equations of the BFM were considered, except for the 3-end mode with the support at the permeate side for the evaluation of flux.

### 3.1. Relative and Absolute Flux Differences between the Different Pressure Simplifications and the Exact Solution

The exact solution describes more realistically the transport through the porous support and will be considered as a reference point for the other simplifications in the further calculations. The effect of varying the transport terms present in the BFM for the exact solution was mapped to the constant pressure simplifications. Finally, the absolute and the relative difference between the different pressure simplifications were investigated using:

(10) $$\mathrm{Relative}\;\mathrm{difference}\;=\frac{N_{\mathrm{CP}}-N_{\mathrm{ES}}}{N_{\mathrm{ES}}}$$

(11) $$\mathrm{Absolute}\;\mathrm{difference}\;=N_{\mathrm{CP}}-N_{\mathrm{ES}}$$

where *N*_CP_ and *N*_ES_ are the fluxes observed from the constant pressure simplifications (averaged constant and surface constant) and the exact solution, respectively. In Table 1, the three transport terms in the BFM that govern the overall transport through the porous support were introduced. The visualization of the three different transport terms of the BFM and the outcome of these variations in a 2-dimensional plot is not possible. Therefore, the transport terms were then combined to represent all effects. The ratio of the viscous flow transport to the binary diffusion transport of the BFM was shown as the vertical axis, while the ratio of the Knudsen diffusion to the binary diffusion transport was the horizontal axis. As a result, by varying the viscous flow, the Knudsen diffusion, and the binary diffusion, the effect of the variation is observed in the horizontal, vertical, and diagonal axes, respectively. The binary diffusion transport term was used as a common denominator since it is less dependent on the microstructure of the porous structure. The axes were combined in this way so that we have all the transport governing terms of the BFM in the map. For each map of the exact solution and the constant pressure simplifications, the dots show the behavioral trend of varying the pore diameter ranging from 10 nm to 50 µm with the present operating parameter. Also for each pore diameter considered, the effect of temperature (the slightly diagonal lines) and tortuosity factor was evaluated.

The tortuosity factor did not show any different effect on the maps when it was varied, because all three transport terms are equally dependent on the tortuosity factor (∝1/k). Thus, its effect cancels out considering the ratios of the term used in the axes.

#### 3.1.1. Support at the Feed Side

From Figure 5, no relative or absolute difference between the exact solution and the averaged constant pressure simplification was observed within the operating conditions (pore diameter ≥ 1 µm and temperature ≥ 900 °C) considered. Within the temperature range considered for pore diameter above 1 µm, the averaged constant pressure simplification can be used in place of the exact solution. However, below 1 µm at reduced temperature, it shifts towards the undesired zone with deviations. The deviation is less than 0.06 mL/min cm^2^ for explicitly given pore diameters even at room temperature. The zone with the highest deviation cannot be reached under relevant physical parameters for the averaged constant pressure simplification.

The comparison between the exact solution and the surface constant pressure simplification shows a higher deviation than the averaged constant pressure simplification. The relative and absolute difference compared between the exact solution and surface constant pressure simplification (Figure 6) shows that the small pore diameters (10 nm–0.1 µm) have the least deviations, although, they are not feasible for the microstructure of the porous structure needed. From Figure 6a, 0.1 µm ≤ pore diameters ≤ 1 µm have a dependence on temperature, while the rest of the pore diameter ranges considered are not dependent on temperature.

For pore diameters greater than 6.5 µm (Figure 6b), the deviation in flux observed for the range of temperatures considered was 0.45 mL/min cm^2^ but was reduced to zero for 10 nm sized pores.

#### 3.1.2. Support at the Permeate Side

Since only the mixed gas permeation was considered for the mapping of the different pressure simplifications, the map of the 3-end mode with the support at the permeate side will not be presented.

When the support is at the permeate side for the 4-end mode, the relative and the absolute difference between the averaged constant pressure simplification and the exact solution (Figure 7) shows that pore diameters smaller than 1 µm were in the zones with least deviation for the operating conditions considered. This was found to be the case for temperatures greater than 900 °C, but reducing the temperature below 900 °C introduces a deviation of 1%. The same deviation was observed for pore diameters greater than 6.5 µm (as received microstructure) which is not more than 1%.

The relative difference between the exact solution and the surface constant pressure simplification for the 4-end mode with the support on the permeate side was observed to be less than ~2% for pore diameters less than 1 µm, but increased up to 3% for pore diameters greater than 1 µm. The absolute difference was observed to be ~0.18 mL/min cm^2^ for pore diameters larger than 6.5 µm and was reduced to 0.02 mL/min cm^2^ for pore diameters less than 0.1 µm.

The large deviations observed for the surface constant pressure simplification when compared to the deviations observed for the averaged constant pressure, makes the latter a better replacement for the implementation of the binary friction model in 3D computational fluid dynamics calculations.

## 4. Conclusions

The optimization of the porous support for different technological applications to improve the overall flux has been an important topic for researchers. For example, the advancement in membrane technology will further be improved if the limitation introduced by the porous support of an asymmetric membrane is brought to the lowest minimal. The exact solution of the BFM gives a realistic description of the transport through the porous structure, but because of the pressure profile, it is complicated to implement in 3D. As a result, the BFM which is used for the description of the transport through the porous structure was simplified with respect to the pressure profiles. Surface constant and averaged constant pressure simplifications of the pressure profile through the porous support were presented and studied. Comparison between the exact and the simplified solutions was shown.

The surface constant and average constant pressure simplifications took 0.20 and 0.27 s for the 1D analytical evaluation of the flux through the membrane. This is more than one order of magnitude less computing time compared to that of the exact solution. For the pressure considered, the absolute difference between surface constant simplification and exact solution for the 3-end mode with the support on the feed side and 4-end mode with the support at the permeate side was 0.45 mL/min cm^2^ and 0.18 mL/min cm^2^, respectively, for a pore size diameter typical of porous support. This reduced with reduced pore diameter, but the small pore sizes are not feasible for the microstructure of the porous support. The relative difference for all transport modes did not increase more than 5%.

For the averaged constant pressure simplification, a deviation of less than 1% from the exact solution for all transport modes, operating assembly, and for a pore size diameter typical of porous support was observed. With the support on the feed side, the deviation reduced to zero for pore diameters above 1 µm and temperatures above 900 °C. This makes the averaged pressure simplification more suitable and it is suggested instead of the exact solution for 3D implementation of the binary friction model.

## Figures and Tables

**Table 1:** The transport governing terms of the binary friction model (BFM) from Equation (7).

Binary Diffusion	Knudsen Diffusion	Viscous Flow
$\frac{P_{j}}{\frac{\varepsilon }{\kappa }D_{\mathrm{ij}}P_{t}}$	$\frac{\varepsilon }{\kappa }D_{g}^{K}$	$\frac{B_{o}P_{g}}{\eta_{g}}$
Dij=10−3T1.75(1Mi+1Mj)12P[(∑Vi)13+(∑Vj)13]2 [19]	$D_{g}^{K}=\frac{d_{\mathrm{pore}}}{3}\sqrt{\frac{8K_{B}T}{\pi M_{g}}}$	$B_{o}\propto d_{\mathrm{pore}}^{2}$

**Table 2:** Input data for the transport equations.

*P_O_*_2_*′* (mbar)	*P_O_*_2_*″* (mbar)	κ	*T* (°C)	*s*_amb_ (S/m)	*d*_pore_ (µm)	*B*_o_ (m^2^)
200	41.5	2.9	900	123.3	6.5	3.09 × 10^−13^
